# Profile, reasons for hospitalization and nursing diagnoses of refugee-native patients admitted to internal medicine clinic-an evaluation from nursing perspective

**DOI:** 10.1186/s12939-024-02190-8

**Published:** 2024-05-09

**Authors:** Neşe Kıskaç, Mahruk Rashidi, Gülay Yıldırım, Abdulkadir Çelik, Burcu Hacıoğlu, Aslı Genç, Sultan Çakmak, Buse Saygın Şahin

**Affiliations:** 1 Faculty of Health Sciences, Department of Nursing, Istanbul Gelişim University, Istanbul, Türkiye; 2 Department of Nursing, Trakya University, Keşan Hakkı Yörük School of Health, Edirne, Türkiye; 3 Haseki Training and Research Hospital, Department of Internal Medicine, Health Sciences University, Istanbul, Türkiye; 4 School of Nursing, Department of Nursing, Ufuk University, Istanbul, Türkiye; 5 Istanbul University- Cerrahpasa/ Institute of Graduate Studies / Mental Health and Diseases Nursing PhD Program, Istanbul, Türkiye

**Keywords:** Diagnosis, Internal medicine, Nursing care, Patient, Temporary refugee

## Abstract

**Purpose:**

The study aims to evaluate the hospitalization diagnoses and nursing diagnoses of the refugee and local population hospitalized in internal medicine clinics, which are especially important in the early diagnosis, treatment, and rehabilitation of chronic diseases, and to emphasize their importance in nursing care.

**Methods:**

The study was carried out in a descriptive retrospective design. The files of 3563 patients admitted to the internal medicine clinic of a training and research hospital in Türkiye in 2022 were evaluated. SPSS 26.0 program was used for data analysis.

**Results:**

In the study, 95.3% of hospitalizations were native and 4.7% were refugee patients. It was determined that refugee patients admitted to the internal medicine service had a lower mean age compared to the native population (*p* < 0.05), but there was no difference in the duration of hospitalization (*p* > 0.05). When the medical diagnoses of hospitalization were examined, it was determined that the highest number of hospitalizations in the native and refugee populations were for bacterial infections in both genders. In nursing diagnoses, it was determined that both populations and genders were diagnosed with infection risk by the medical diagnoses of the patients.

**Conclusion:**

As a result of the study, it was observed that the duration of hospitalization, reasons for hospitalization, and nursing diagnoses of local and refugee patients were similar. In addition, it was determined that the patients’ medical hospitalization diagnoses and nursing diagnoses were compatible.

## Introduction

Internal medicine clinics are units of hospitals that require a holistic and multisystemic approach, where the diagnosis, treatment, follow-up, care, and rehabilitation of non-surgical acute problems and chronic diseases of individuals are performed [1–3]. It is stated that the treatment and care of inpatients in internal medicine clinics constitute a large part of health services, with the prolongation of life expectancy and the increase in chronic diseases in the world and in our country [4]. In their study conducted in Türkiye in 2018, Karahan and Çifci found that the first reason for hospitalization of 303 patients admitted to the internal medicine clinic was the regulation of blood sugar in diabetic patients, the second reason was electrolyte disorders, and the third reason was to investigate the cause of anemia [3]. In their study conducted in Türkiye in 2011, Nalbant et al. examined the most common diagnoses of 316 patients over 65 years of age admitted to the internal medicine clinic and found that 60% (189) of all cases were hospitalized with the diagnosis of anemia, followed by hypertension in 41.3% (130) and diabetes mellitus in 35% (110) [5]. Many factors such as war, epidemics, and natural disasters in the world can change the patient profile and reasons for applying to hospitals. Migrants who had to migrate to other countries due to the wars in the world started to increase in Türkiye after 2011 [6]. Aygün et al. examined the reasons for admission of refugees to a community health center in Türkiye in 2016 and found that the most common reason for admission was infection [7]. Among these migrants, there has been a large increase in the number of Syrian migrants and their health expenditures have been covered by Türkiye [8]. Immigrants, who receive free healthcare services, especially apply to public hospitals and receive healthcare services.

Nurses play an active role in the care of refugee and native populations receiving health services from public hospitals. Understanding the health status of these two different populations and providing effective care requires nurses to have deep knowledge and experience in cultural sensitivity, communication skills, and multidisciplinary collaboration [9]. The evaluation of medical diagnoses of refugee and native populations admitted to internal medicine clinics also points to an area that requires special attention in nursing care, as in all areas. First, assessing the medical diagnoses of refugee and native patients reveals diseases that may be common in both communities, such as chronic diseases, infections, and metabolic disorders. At this point, nurses must be equipped with a wide range of medical knowledge and are familiar with current clinical guidelines.

In conclusion, the assessment of medical diagnoses in refugee and native populations hospitalized in internal medicine clinics is an area that requires special attention in nursing care but also offers the potential for deep satisfaction and learning. This article emphasizes the importance of nursing care in terms of hospitalization diagnoses and nursing diagnoses of the refugee and local population hospitalized in internal medicine clinics, which are especially important in the early diagnosis, treatment, and rehabilitation of chronic diseases.

## Materials and methods

### Study design

The research was conducted in a descriptive retrospective design.

### Sample of the research

No sample selection was made in the study. The files of all patients admitted to the internal medicine clinic of a training and research hospital in Türkiye in 2022 were scanned from the hospital database. A total of 3563 patient files were accessed.

### Data collection method

Data were obtained by querying the hospital database. The files of patients aged 18 years and older who were admitted to the internal medicine clinic in 2022 and started to receive treatment were included in the study. While determining the hospitalization diagnoses, the admission diagnosis in the hospital records and the diagnoses recorded in the file were noted as diagnoses. While evaluating the files, the admission diagnosis was taken as the basis. As a result of the screening, the first 23 hospitalization diagnoses, and 19 nursing diagnoses with the highest number of 3563 patients admitted to the internal medicine clinic, were analyzed. It is made according to the International Classification of Disease. NANDA diagnoses are used in nursing diagnoses.

### Data collection

While collecting the data, the patient file form was used for the data requested to be accessed in the patient file. The patient file form consisted of 6 (six) questions including the patient’s age, gender, nationality, number of days of hospitalization, hospitalization diagnosis, and nursing diagnosis.

IBM SPSS 26.0 statistical program was used in the data analysis of this study. In addition to descriptive statistical methods (mean, median, standard deviation, frequency, percentage), the Student T-test was used to compare normally distributed data, and the Mann-Whitney U test was used to compare non-normally distributed data. Skewness, kurtosis values, and the Shapiro-Wilk test were used to evaluate the normal distribution. The results were evaluated at 95% confidence interval and *p* < 0.05 significance level.

### Study limitations

This study was conducted in a hospital in Istanbul, Türkiye. The barriers and facilitators of refugees’ access to health are influenced by the cultural structure of the region and the opportunities offered by the region. In the study, no difference was detected regarding hospitalization diagnoses in the refugee and native populations. The fact that the study was conducted in a single center is considered to be a limitation. Expanding the study to different regions may offer different perspectives.

### Ethical considerations

Before starting the study, permission was obtained from the Ethics Committee of Haseki Training and Research Hospital with the date June 22, 2023, and decision number 93-2023. Patients’ data was protected while the research data was scanned from the hospital database.

## Results

The files of 3563 patients admitted to the internal medicine clinic in 2022 were reviewed retrospectively. As a result of the scan, 3395 patients (95.3%) were native, 168 (4.7%) patients were refugees, 50% of native patients were women, 50% were men, 57.1% of refugee patients were women, 42.9% were men, and it was determined that the average age of native patients was 63.09 ± 17.39, mean hospitalization time was 5.09 ± 4.87, mean age of refugee patients was 49.23 ± 16.32, mean hospitalization time was 4.74 ± 4.96. Considering the nationalities of the patients hospitalized in the internal medicine clinic, it was determined that 95.3% were Türkiye, 3.6% were Syrian, and 1.1% were refugees outside Syria (Table 1).

**Table 1:** Personal characteristics of patients admitted to the internal medicine clinic (*n* = 3563)

		Native (*n* = 3395)		Refugee(*n* = 168)	
		*n*	%	*n*	%
**Nationality**					
**Türkiye**		3395	95.3	0	0
**Syria**		0	0	127	3.6
**Others**		0	0	41	1.1
**Gender**					
**Female**		1697	50	96	57.1
**Male**		1698	50	72	42.9
**Age (years)**	**mean**	63.09 ± 17.39		49.23 ± 16.32	
	**median**	66		49	
**Duration of hospitalization (days)**	**mean**	5.09 ± 4.87		4.74 ± 4.96	
	**median**	4		3	

Descriptive statistical methods (mean, median, standard deviation, frequency, percentage)

The significance between the refugee and native population in terms of age and duration of hospitalization was examined according to gender. A significant difference was found between the mean ages of the two populations in both genders (*p* = 0.001; *p* < 0.05), and no significant difference was found between the duration of hospitalization (*p* > 0.05) (Table 2).

**Table 2:** Distribution of refugee-native populations by gender in terms of age and length of stay

		Refugee		Native		p
		mean	median	mean	median	
**Age of patient (Years)**	**Female**	50.72 ± 16.54	49	64.50 ± 17.93	69	0.001
	**Male**	47.25 ± 15.91	50	61.68 ± 16.71	63	0.001
**Duration of hospitalization** **(Days)**	**Female**	4.44 ± 5.19	2	5.26 ± 5	4	0.118
	**Male**	5.15 ± 4.62	4	4.92 ± 4.74	3	0.681

Mean, median, Student T test, Mann-Whitney U test

The hospitalization diagnoses of patients admitted to the internal medicine clinic in 2022 were examined from their files and the 23 most common diagnoses were evaluated. Considering the hospitalization diagnoses in the native population, bacterial infection (65.6%) is the first in women, vitamin D deficiency (60.2%) is the second, Type 2 diabetes is the third (49.3%), bacterial infection is the first (68.3%) in men, and vitamin D deficiency is the second (62%) and Type 2 diabetes (51.7%) ranked third. Considering the hospitalization diagnoses of the refugee population, bacterial infection (70.8%) is the first in women, vitamin D deficiency (56.3%) is the second, Type 2 diabetes is the third (49%), bacterial infection is the first (66.7%) in men, and vitamin D deficiency is the second (58.3%) and Type 2 diabetes (43.1%) were the third (Table 3). When the hospitalization diagnoses of the patients are evaluated in their entirety, bacterial infection is the first most common reason for hospitalization in both native and refugee populations and in both genders, vitamin D deficiency is the second most common reason and Type 2 diabetes is the third most common reason (Table 3).

**Table 3:** Distribution of patients admitted to the internal medicine clinic according to hospitalization diagnosis (native female *n* = 1697; native male *n* = 1698; refugee female *n* = 96; refugee male *n* = 72)

Diagnosis	Gender	Native		Refugee		Diagnosis	Gender	Native		Refugee	
		n	%	n	%			n	%	n	%
Acute Kidney Failures	**Female**	505	29.8	41	42.7	Heart failure	**Female**	44	2.6	8	8.3
	**Male**	591	34.8	24	33.3		**Male**	90	5.3	0	0
Anemia	**Female**	300	17.6	4	4.2	Nausea and Vomiting	**Female**	185	10.9	8	8.3
	**Male**	226	13.3	15	20.8		**Male**	160	9.4	11	15.3
Bacterial infection	**Female**	1114	65.6	68	70.8	Peripheral Vascular Disease	**Female**	36	2.1	1	1.0
	**Male**	1160	68.3	48	66.7		**Male**	67	3.9	0	0
Chest Pain	**Female**	102	6.0	7	7.3	Pneumonia	**Female**	148	8.7	9	9.4
	**Male**	80	4.7	2	2.8		**Male**	184	10.8	9	12.5
Chronic obstructive pulmonary disease	**Female**	70	4.1	1	1.0	Protein-Energy Malnutrition	**Female**	168	9.9	5	5.2
	**Male**	193	11.4	1	1.4		**Male**	210	12.4	6	8.3
Chronic Respiratory Failure	**Female**	134	7.9	11	11.5	Stomach ache	**Female**	324	19.1	21	21.9
	**Male**	118	6.9	2	2.8		**Male**	338	19.9	22	30.6
COVID-19	**Female**	201	11.8	6	6.3	Type 1 Diabetes	**Female**	135	8.0	5	5.2
	**Male**	170	10.0	9	12.5		**Male**	97	5.7	6	8.3
Diseases of the Blood and Blood-Making Organs	**Female**	189	11.1	10	10.4	Type 2 Diabetes	**Female**	836	49.3	47	49.0
	**Male**	203	12.0	14	19.4		**Male**	878	51.7	31	43.1
Dyspnea	**Female**	286	16.9	6	6.3	Urinary System Infection	**Female**	108	6.4	13	13.5
	**Male**	208	12.2	22	30.6		**Male**	304	17.9	7	9.7
End Stage Kidney Disease	**Female**	304	17.9	17	17.7	Viral Diseases	**Female**	85	5.0	1	1.0
	**Male**	456	26.9	11	15.3		**Male**	97	5.7	1	1.4
Essential (Primary) Hypertension	**Female**	115	6.8	5	5.2	Vitamin D Deficiency	**Female**	1021	60.2	54	56.3
	**Male**	65	3.8	1	1.4		**Male**	1052	62.0	42	58.3
Gastrointestinal Hemorrhage	**Female**	107	6.3	9	9.4						
	**Male**	158	9.3	8	11.1						

Descriptive statistical methods (frequency, percentage)

Nineteen of the most common nursing diagnoses for patients admitted to the internal medicine clinic in 2022 were examined. Among the nursing diagnoses for local patients, the risk for infection ranked first in women (86.6%), the risk for falls ranked second (82.9%), and deficient knowledge ranked third (56.9%), while the risk for infection ranked first in men (87.3%), the risk for falls ranked second (82.1%), and deficient knowledge ranked third (57.8%). When the nursing diagnoses made for refugee patients are analyzed, it is seen that the risk for infection ranks first (91.7%), the risk for falls ranks second (81.3%), and the deficient knowledge ranks third (69.8%) in women, and the risk for infection ranks first (93.1%), the risk for falls ranks second (76.4%), and the deficient knowledge ranks third (69.4%) in men.

**Table 4:** Distribution of nursing diagnoses made in patients hospitalized in the internal medicine clinic (native female *n* = 1697; native male *n* = 1698; refugee female *n* = 96; refugee male *n* = 72)

Diagnosis	Gender	Native		Refugee		Diagnosis	Gender	Native		Refugee	
		n	%	n	%			n	%	n	%
Acute Kidney Failures	**Female**	505	29.8	41	42.7	Heart failure	**Female**	44	2.6	8	8.3
	**Male**	591	34.8	24	33.3		**Male**	90	5.3	0	0
Anemia	**Female**	300	17.6	4	4.2	Nausea and Vomiting	**Female**	185	10.9	8	8.3
	**Male**	226	13.3	15	20.8		**Male**	160	9.4	11	15.3
Bacterial infection	**Female**	1114	65.6	68	70.8	Peripheral Vascular Disease	**Female**	36	2.1	1	1.0
	**Male**	1160	68.3	48	66.7		**Male**	67	3.9	0	0
Chest Pain	**Female**	102	6.0	7	7.3	Pneumonia	**Female**	148	8.7	9	9.4
	**Male**	80	4.7	2	2.8		**Male**	184	10.8	9	12.5
Chronic obstructive pulmonary disease	**Female**	70	4.1	1	1.0	Protein-Energy Malnutrition	**Female**	168	9.9	5	5.2
	**Male**	193	11.4	1	1.4		**Male**	210	12.4	6	8.3
Chronic Respiratory Failure	**Female**	134	7.9	11	11.5	Stomach ache	**Female**	324	19.1	21	21.9
	**Male**	118	6.9	2	2.8		**Male**	338	19.9	22	30.6
COVID-19	**Female**	201	11.8	6	6.3	Type 1 Diabetes	**Female**	135	8.0	5	5.2
	**Male**	170	10.0	9	12.5		**Male**	97	5.7	6	8.3
Diseases of the Blood and Blood-Making Organs	**Female**	189	11.1	10	10.4	Type 2 Diabetes	**Female**	836	49.3	47	49.0
	**Male**	203	12.0	14	19.4		**Male**	878	51.7	31	43.1
Dyspnea	**Female**	286	16.9	6	6.3	Urinary System Disease	**Female**	108	6.4	13	13.5
	**Male**	208	12.2	22	30.6		**Male**	304	17.9	7	9.7
End Stage Kidney Disease	**Female**	304	17.9	17	17.7	Viral Diseases	**Female**	85	5.0	1	1.0
	**Male**	456	26.9	11	15.3		**Male**	97	5.7	1	1.4
Essential (Primary) Hypertension	**Female**	115	6.8	5	5.2	Vitamin D Deficiency	**Female**	1021	60.2	54	56.3
	**Male**	65	3.8	1	1.4		**Male**	1052	62.0	42	58.3
Gastrointestinal Hemorrhage	**Female**	107	6.3	9	9.4						
	**Male**	158	9.3	8	11.1						

Descriptive statistical methods (frequency, percentage)

## Discussion

Türkiye is the country that has received the most refugees for nine consecutive years. Health policies have been developed in our country to protect and improve the health of refugees and to facilitate their access to health services. It is thought that it may lead to changes in the hospitalization profiles and hospitalization diagnoses of refugees benefiting from health services in our country. This study evaluates the profile and reasons for hospitalization of refugee-native patients admitted to the internal medicine clinic.

In the study, it was determined that 95.3% of the patients admitted to the internal medicine service were native patients and 4.7% were refugees (Table 1). According to the data of the Ministry of Interior General Directorate of Migration Management in our country, it was determined that the number of Syrians under temporary protection in 2023 was 3.411.029 and this ratio constituted approximately 4.5% of the population of Türkiye [10]. In the research, it was seen that Syrian nationals constitute the most migration among the refugees, and the rate of refugees who applied to the internal medicine service was similar to the population rate of Türkiye, according to the data of the General Directorate of Migration Management. If the migration from Syria continues, it is thought that the hospitalization rates of refugees will increase.

In the study, it was determined that 3.6% of the refugees were Syrian nationals (Table 1). The United Nations High Commissioner for Refugees (UNHCR) emphasized that the population of Syria was 22.5 million in 2011, 13.5 million of them were forcibly displaced according to 2020 data [11], and more than 80% of the migrations were made to neighboring countries mainly Türkiye, Lebanon and Jordan [12]. In our country, the General Directorate of Migration Management of the Ministry of Internal Affairs stated that the most migration to Türkiye is from Syria, Afghanistan, and Iraq [13, 14]. It is an expected result that Syrian nationals are the most refugees among the refugees in the research.

In the study, it was found that refugee patients admitted to the internal medicine service had a lower mean age than the native population (*p* < 0.05) (Table 2). When the demographic characteristics of the Syrian refugees in Türkiye are examined, it has been emphasized that 85% of the Syrian population living in our country is a young population under the age of 40 [13]. The fact that the majority of refugee women and men in the study are Syrians and the young population of Syrians who migrated to our country may be the reason why refugee women and men have a lower average age.

Considering the hospitalization diagnoses, it was determined that both the native and refugee population’s men and women were diagnosed with bacterial infection in the first place (Table 3). 1,299,209 cases of respiratory tract infections were reported in Syrian refugees living in temporary shelters between 2012 and 2016 [15]. In another study examining the access and use of health services by Syrian refugees in our country, it was emphasized that Syrian refugees living in camps pose a public health threat to the population they live in [14]. The majority of Syrian refugees in Türkiye live in camps, while others live in neighborhoods with low socio-economic status. It is thought that refugees often apply to the hospital with the diagnosis of bacterial infection since several families living together in unhygienic, crowded environments will cause more frequent infectious diseases and transmission. When we look at the studies in the literature, it is seen that refugee patients are mostly monitored in terms of mental health [16–18]. In the study conducted by Raphael et al. in the USA, it was found that there were differences between refugee patients and patients born in the USA in terms of disease diagnoses. Patients born in the USA were diagnosed more frequently with chronic diseases and less frequently with mental health diseases compared to refugee patients [19]. In a study conducted by Odeh et al. with refugee Type 1 diabetic children in Jordan, it was found that children had low metabolic control and frequent hypoglycemia [20]. In the study conducted in Türkiye, there was no difference between the hospitalization diagnoses of native patients and refugee patients, whereas, in other countries, there were differences between the two groups in terms of disease diagnoses. It is thought that this difference may be caused by the differences in the health policies of the countries and the opportunities provided to refugee individuals such as health, education, transportation, and shelter.

In the study, it was determined that the nursing diagnoses determined for the patients in both populations and genders were risk for infection in the first place, risk for fall in the second place, and deficient knowledge in the third place (Table 4). In a study conducted by Korhan et al. (2014) with intensive care patients, it was determined that the most commonly used nursing diagnosis was Impaired skin integrity, deterioration in oral mucous membrane, and deterioration in verbal communication [21]. In a study conducted by Santos et al. (2023) in Brazil, it was determined that the most commonly determined nursing diagnoses in patients were risk for infection, risk for falls, and risk of pressure sores [22]. While the results of the study conducted by Santos et al. and this study are similar, they are different from the study conducted by Korhan et al. We can say that the difference between the results of the study conducted by Korhan et al. and this study is that the sample group in the study conducted by Korhan et al. was intensive care patients.

In this study, it was observed that the medical hospitalization diagnoses and nursing diagnoses of refugee and native patients hospitalized in the Internal Medicine Clinic were compatible. While the highest medical hospitalization diagnosis was a bacterial infection, it was determined that there was a risk of infection in the nursing diagnosis. Again, when we look at the medical hospitalization diagnoses of the patients, we see that they have chronic diseases and symptoms. Individuals with chronic diseases experience frequent hospitalizations when they do not receive adequate education and information. The nurses’ other frequent diagnoses of fall risk and lack of information are consistent with the patient groups hospitalized in internal medicine.

Cultural diversity can increase the complexity of these assessments. Refugee patients may often have different language and cultural backgrounds. Nurses must become experts in overcoming language barriers and developing cultural sensitivity to overcome these challenges and communicate effectively with the patient. Understanding patients’ cultural beliefs is critical when creating and implementing treatment plans.

On the other hand, the refugee population may require specialized care, often due to traumatic events they have experienced in the past. Nurses should be sensitive enough to identify patients’ emotional needs, provide psychosocial support, and seek specialized help if necessary.

When evaluating the medical diagnoses of these two populations, it should be kept in mind that nurses play an important role in primary health care. Nurses should provide customized care for individual needs through patient education, health promotion, and leadership in creating patient care plans.

## Conclusion

As a result of the study, it was observed that the duration of hospitalization, reasons for hospitalization, and nursing diagnoses of local and refugee patients were similar. In addition, it was determined that the patient’s medical hospitalization diagnoses and nursing diagnoses were compatible. According to this result, it is thought that nursing diagnoses and care are made by considering cultural differences and medical diagnoses of patients.

## Data Availability

No datasets were generated or analysed during the current study.
